# Developing Problem-Solving Expertise for Word Problems

**DOI:** 10.3389/fpsyg.2022.725280

**Published:** 2022-05-03

**Authors:** Bing Hiong Ngu, Huy P. Phan

**Affiliations:** School of Education, University of New England, Armidale, NSW, Australia

**Keywords:** problem-solving, expertise, worked examples, word problems, cognitive load

## Abstract

Studying worked examples impose relatively low cognitive load because learners’ attention is directed to learn the schema, which is embedded in the worked examples. That schema encompasses both conceptual knowledge and procedural knowledge. It is well-documented that worked examples are effective in facilitating the acquisition of problem-solving skills. However, the use of worked examples to develop problem-solving expertise is less known. Typically, experts demonstrate an efficient way to solve problems that is quicker, faster, and having fewer solution steps. We reviewed five studies to validate the benefit of worked examples to develop problem-solving expertise for word problems. Overall, a diagram portrays the problem structure, coupled with either study worked examples or complete multiple example–problem pairs, facilitates the formation of an equation to solve words problems efficiently. Hence, an in-depth understanding of conceptual knowledge (i.e., problem structure) might contribute to superior performance of procedural knowledge manifested in the reduced solution steps.

## Introduction

Do we need to teach students how to develop expertise in mathematical problem solving? If so, how can mathematics educators accomplish such a goal? To explore how experts solve linear equations, Star and Newton (2009) interviewed eight experts in the domain of algebra (e.g., mathematics teachers). Regarding a linear equation, such as 7(*n* + 13) = 42, one expert viewed the division by 7 on both sides as the first step would give “a nice, clean answer.” Another expert commented on an inefficient way of using distribution to remove the bracket as a first step—“Distributing, I would have had to deal with fractions and finding common denominators and things would not have been as nice” (p. 6). The findings indicate that the solution produced by experts is typically fewer steps, faster, and quicker.

Within the framework of cognitive load theory, the objective of the present paper was to review prior studies (Ngu et al., 2009, 2014, 2016, 2018; Ngu and Yeung, 2013) to examine the impact of worked examples upon the development of problem-solving expertise for word problems. More specifically, we examined students’ solution strategies to determine evidence of problem-solving expertise. Across five studies, we attributed students’ ability to use a single equation to solve a category of word problems with fewer solution steps as the demonstration of problem-solving expertise. Furthermore, evidence of problem-solving expertise also includes an adaption of the solution procedure of similar problems to solve transfer problems that differ slightly from similar problems in terms of problem structure. We will begin by discussing differential performance between experts and novices in different domains.

## Differential Performance Between Experts and Novices

The seminal work of De Groot (1965) uncovered differential knowledge base of chess configurations between expert and novice chess players. When presented with a specific chess configuration, the expert chess players relied on schemas that contained thousands of chess configurations to guide the best move. In contrast, novices lacked appropriate schemas related to chess configurations to guide the best move. The findings suggest that expertise resides in having schemas, which contain domain-specific knowledge for a domain (Tricot and Sweller, 2014).

According to Chi et al. (1981), experts categorized physics word problems in accord with a specific principle (e.g., conservation of energy). Novices, on the other hand, categorized physics word problems based on surface features (e.g., inclined planes). In mathematics domain, experts were capable of categorizing a group of word problems based on the underlying principle or shared problem structure (Silver, 1979). Clearly, the findings by Chi et al. (1981) and Silver (1979) suggest that the presence of schemas differentiates problem-solvers’ levels of knowledge and therefore levels of problem-solving expertise.

More recent research has shifted emphasis on the acquisition of both conceptual and procedural knowledge as evidence of mathematical proficiency (Rittle-Johnson et al., 2001; Baroody et al., 2007). The conceptual knowledge refers to knowledge of the underlying principle that connects interrelated mathematical concepts for a specific topic, whereas procedural knowledge refers to the application of a sequential actions to obtain the solution (Rittle-Johnson et al., 2001). Hiebert and Lefevre (1986) suggested that competence in conceptual knowledge assists in the execution of procedural knowledge. A review by Bethany Rittle-Johnson et al. (2015) indicated bidirectional relationship between conceptual knowledge and procedural knowledge. The gaining of conceptual knowledge facilitates the gaining of procedural knowledge and vice versa. Accordingly, we would expect an expert in mathematics domain to possess a schema that would reflect competence in both conceptual knowledge and procedural knowledge specific to a topic.

Blessing and Anderson (1996) examined how learners skipped steps after having acquired algebraic rules to solve problems. Apparently, once novice learners became expert learners, they could recognize a specific pattern that allowed them to skip intermediate steps mentally and create fewer solution steps. An advantage of step skipping performance is that it permits expert learners to solve problems more easily, quickly and efficiently. Likewise, the experts in geometry proof problems could infer from the diagram the whole statement schema related to a geometrical shape (e.g., congruent triangles-shared side; Koedinger and Anderson, 1990). Then, they used a minimal number of identical angles and sides to proof that the two triangles are congruent. In short, the step skipping performance exhibited by experts reflects the presence of a schema pertaining to conceptual knowledge and procedural knowledge. Presumably, that schema allows the experts to use conceptual knowledge to refine the solutions steps, resulting in fewer solution steps.

Mayer (1985) suggested that the presence of schematic knowledge is critical to success in solving word problems. Drawing on their schematic knowledge, the problem-solvers could select values and variable from the problem text, and integrate these in an equation for solution. In other words, the ability to identify structural elements (values, variable) from problem text and express these in an equation reflects the availability of a schema for a category of problems. For example, one can use a *t*-test to solve a category of statistic word problems that share a similar problem structure (Quilici and Mayer, 1996). Indeed, differential ability to construct a mathematics-specific equation to solve word problems is a critical factor that differentiates successful and unsuccessful problem-solvers (Hegarty et al., 1995). Successful problem-solvers were more likely than unsuccessful problem-solvers to construct a mathematics-specific equation for generating a solution. Hence, the ability to match a problem with a known solution path, and use a single equation to solve word problems is regarded as the demonstration of problem-solving expertise (Blessing and Ross, 1996). While past research has identified superior performance of successful problem-solvers on word problems, addressing the issue of developing problem-solving expertise for word problems is less clear.

Research has examined how novices become experts across diverse domains (Ericsson, 2006). Apparently, a substantial length of 10 years of practice is required to develop expertise across a range of domains, such as music, sports, and so on. Ericsson (2006) has recommended the use of deliberate practice to gain expertise in a domain. Specifically, the deliberate practice activities target a learner’s weakness of a particular aspect of the subject matter. For example, the deliberate practice activities requiring students to calculate the area of geometrical shapes (which was identified as a weak area of students) had improved their performance on geometry problems (Pachman et al., 2013). Apart from the study by Pachman et al. (2013), there is limited research investigating the development of problem-solving expertise for word problems. We argue that learning with the aid of worked examples can address such an issue. Because the use of worked examples to enhance mathematics learning is one of the cognitive load effects, we will discuss the theoretical rationale of cognitive load theory and instructional design in the next section.

## Cognitive Load Theory

Cognitive load theory is an instructional theory that has influenced the design of instructions across diverse domains (Sweller et al., 2011). The NSW Department of Education has advocated teachers to examine evidence-based research generated by cognitive load theory (e.g., worked examples) to improve instructional practices in different disciplines, and one of which is mathematics education (NSW Education: Centre for Education Statistics and Evaluation, 2017). This study will review evidence-based research to support the use of worked examples to enhance problem-solving skills for word problems across mathematics and chemistry curriculum.

Cognitive load theory emphasizes the alignment between human cognitive architecture and instructional design to facilitate learning. The human cognitive architecture has a long-term memory that provides a huge storage for knowledge structure in the form of schemas. Most of the schemas are obtained from the long-term memory of other people. Early work by Miller (1956) indicated that it also has a limited working memory that can process seven elements at any given time, but more recent research indicates that it can process about four elements (Cowan, 2001). Furthermore, information readily disappears without being rehearsed (Peterson and Peterson, 1959). Once the information has been processed successfully in the working memory, it will be stored in the long-term memory in the form of schemas.

Cognitive load theory distinguishes three types of cognitive load (intrinsic, extraneous, germane). The intrinsic cognitive load is imposed by the complexity of materials that in turn, is governed by the level of element interactivity that a task contains. The level of element interactivity is determined by the interaction between elements, which must be processed simultaneously to allow understanding to occur. An element refers to anything that requires to be learned (e.g., a number, symbol, and a procedure; Chen et al., 2017). The intrinsic cognitive load depends on the complexity of the materials, and learners’ expertise level. The intrinsic cognitive load imposed on working memory increases as the level of element interactivity of the task increases. However, once novices gain expertise, they can “chunk” multiple interactive elements into a schema and store this in the long-term memory. Because we can process the schema retrieved from the long-term memory as a single entity in the working memory, it reduces the intrinsic cognitive load imposed on the working memory. Hence, the limitation of the working memory occurs when processing novel information, but not schemas from the long-term memory. From the perspective of expertise development, an automated schema (Cooper and Sweller, 1987) will free up working memory to allow problem-solvers to deal with aspects of the transfer problem that are unfamiliar.

The extraneous cognitive load is imposed by inappropriate instructional designs that are ineffective for learning. Hence, extraneous cognitive load should always be eliminated by instructional designers. For example, in the domain of geometry problems, splitting learners’ attention between the diagram and the solution steps causes a split-attention effect, imposing extraneous cognitive load that impairs learning (Tarmizi and Sweller, 1988). We can eliminate the split-attention effect by placing individual solution steps at relevant positions in the diagram.

The germane cognitive load is evoked by appropriate instructional designs that are effective for learning. More recent development of cognitive load theory suggests that germane cognitive load does not impose an independent cognitive load. Rather, it is part of the intrinsic cognitive load given that a learner invests germane cognitive load to understand the intrinsic nature of the task (Sweller, 2010). The design of the variability practice increases germane cognitive load, but it benefits learning (Paas and Van Merriënboer, 1994; Likourezos et al., 2019). For example, under the variability practice condition, learners are expected to invest germane cognitive load to identify a shared problem structure across a category of problems that differs in problem contexts. As will be discussed later, the provision of a diagram that depicts conceptual knowledge (problem structure) of percentage problems increases germane cognitive load and thus it contributes toward learning (Ngu et al., 2014, 2018).

The three types of cognitive load (intrinsic, extraneous and germane) have implication for designing effective instructions. To optimize the acquisition of problem-solving skills, we need to minimize extraneous cognitive load, optimize germane cognitive load and to ensure that the intrinsic cognitive load of the material is appropriate for learners. One such effective instructional method is the use of worked examples, which has been demonstrated across multiple studies (Sweller et al., 2011).

### The Worked Example Effect

One of the most widely researched cognitive load effects is the worked example effect. The worked example effect occurs when studying worked examples resulted in better learning outcomes and imposes lower cognitive load than solving the same problems particularly for novices in a domain (Sweller et al., 2011; Renkl, 2014). A worked example provides detailed solution steps to solve a problem. The solution steps of a worked example encompass a schema required to solve a category of problems. Such a schema is regarded as domain-specific knowledge for a category of problems that share a similar problem structure (Tricot and Sweller, 2014).

According to cognitive load theory, studying worked examples allow learners to focus on an understanding of the relation between problem states and problem-solving operators (e.g., algebra rules; Sweller and Cooper, 1985). Accordingly, studying worked examples impose low cognitive load and thus facilitates schema acquisition. In other words, studying worked examples represents an efficient way to overcome the limitation of working memory resources. In contrast, problem-solving approach imposes extraneous cognitive load because cognitive resources are used to search for a solution path, which interferes with the acquisition of schema. As highlighted in a review by Gog et al. (2019), practice problem-solving compels learners to search for a solution procedure, which not only consumes a lot of cognitive resources but also time (McLaren et al., 2016) and thus is not an efficient way to acquire schema. In essence, the worked example effect relies on the “borrowing and reorganizing principle” of information processing (Sweller, 2010; Chen et al., 2015). It makes senses to borrow the schemas from the long-term memory of experts in a domain instead of using cognitive resources to search for a solution path as in the case of problem-solving approach.

Since the inception of cognitive load theory more than three decades ago, empirical studies that support the worked example effect across different domains are overwhelming (Sweller et al., 2011). Early work on the worked example effect was found in learning algebra transformation problems (Sweller and Cooper, 1985). Other studies in relation to the worked example effect are found in statistics (Paas, 1992), physics (van Gog et al., 2008), chemistry (Ngu et al., 2009), and geometry (Tarmizi and Sweller, 1988; Bokosmaty et al., 2015). Recently, researchers has extended the worked example effect to learn how to write Chinese characters in which each character consists of various components (Lu et al., 2020). Presenting isolated component of each Chinese character in a variable format was more helpful for novice learners than the blocked format. The variable format allows novice learners to practice variable components of a Chinese character consecutively instead of a uniform component as in the case of a blocked format. In light of a volume of worked examples research, one may wonder how did researchers implement the worked example effect?

### Implementation of Worked Examples

Research has found that students may merely look at worked examples rather than paying attention to the worked-out solution steps of worked examples (Reed et al., 1985). Without paying attention to the solution steps, it is unlikely that learners can abstract a schema that is embedded in worked examples, and then use this to solve similar problems. Renkl and his colleagues have advocated the incorporation of prompts or self-explanation (Renkl, 1999; Schworm and Renkl, 2006) to help learners focus on the underlying concepts embedded within worked examples. In order to prevent students from studying worked examples superficially, Sweller and Cooper (1985) required students to study a worked example paired with a problem. They reasoned that students would be more motivated to study a worked example, if they knew that they needed to solve a similar problem after studying the worked example. This pioneer work of studying a worked example paired with a problem becomes a blue print for effective implementation of the worked example effect (Sweller et al., 2011). As attested by the findings of Trafton and Reiser (1993), requiring learners to study a block of six worked examples, followed by solving a block of six problems was less effective than “study-one and solve-one” strategy. Indeed, more recent research has compared study examples only, example-problem pairs, problem–example pairs and problem-solving (van Gog et al., 2011). Unsurprisingly, study examples only and complete example–problem pairs were better than either problem–example pairs or problem-solving only in terms of investing less effort in the acquisition phase and achieve better learning outcomes. The authors argued that novice learners, in particular, may not be able to diagnose their own errors in the solution. Therefore, problem–example pairs condition in which novice learners solved a problem paired with a worked example (which can act as feedback) may not be helpful. Of the five studies that we will discuss, one study uses study examples only and the other four studies use example–problem pairs. Importantly, across five studies, both studying worked examples only and completing example–problem pairs serves as direct instruction to facilitate the development of problem-solving expertise. In relation to word problems, it is important to know the types of knowledge required to solve word problem and the role of a diagram to enhance learning of word problems. We will discuss both of these in the next section.

## Importance of a Diagram

According to Mayer (1982), five types of knowledge are needed for solving word problems: linguistic, factual, schematic, strategic and algorithmic. Knowledge of the linguistic, factual and/or numerical components enables problem-solvers to translate and understand problem situation. The schematic knowledge enables problem-solvers to classify a problem with respect to a category of problems and the manner in which the problem can be solved. The strategic and algorithmic knowledge enables problem-solvers to plan a solution procedure for solving word problems.

Mayer suggested that the greatest hurdle for solving word problems is to represent word problems amenable for generating solutions. Prior studies have demonstrated the power of a diagram to represent word problems by displaying the relationship among quantitative values and variable in order to aid in the construction of a mathematical relationship for solutions (Mayer and Gallini, 1990; Hegarty and Kozhevnikov, 1999; Ng and Lee, 2009; Schwonke et al., 2009; Zahner and Corter, 2010; Jitendra et al., 2011). However, the design of a diagram matters. As revealed by Hegarty and Kozhevnikov (1999), schematic diagrams displaying spatial information that captures the problem structure facilitates higher solution success than the non-schematic diagrams (pictorial diagrams) that illustrate cover stories. In other words, a schematic diagram can assist a learner to translate abstract relationships within the problem text and make it concrete. Indeed, using a schema-based instruction incorporating a diagram, which shows mathematical relationship (values and variable) cited in the problem text has improved learning of word problems (e.g., Jitendra et al., 2011). Accordingly, of the five studies that we reviewed, four studies incorporate diagrams that seek to capture the problem structure of word problems. For example, the equation approach in Study 3 and Study 4 has a horizontal line in which a shorter length of this line represents a fraction of a percentage quantity. Furthermore, in our review, three out of the five studies involve cross-cultural mathematics education—we will discuss this in the next Section.

## Cross-cultural Mathematics Education

Research has indicated that students from different cultural backgrounds (China vs. U.S) use different approaches (algebra vs. non-algebra) to solve word problems (Cai, 2000). The Chinese students tended to use the algebra approach, which requires the formulation of an equation, and then solve for the unknown variable, *x*. On the other hand, U.S students preferred the use of non-algebra approach, which may rely on concrete visual representations (e.g., drawing a picture) to solve word problems. Regarding the content knowledge of solving linear equations, Ngu and Phan (2020) found that Australian pre-service teachers were inferior to Malaysian pre-service teachers. An analysis of primary mathematics education curriculum reveals that Asian countries (e.g., China, Korea, and Japan) have introduced the topic of linear equations in primary mathematics curriculum, but not Western countries (Cai et al., 2005). Hence, an earlier exposure to algebra may have helped Asian students to build a stronger algebra foundation than their peers in Western countries. In the current review of five studies, we included cross-cultural studies to highlight differential performance between Australian students and Malaysian students especially in regard to the use of algebra in gaining problem-solving expertise for word problems.

## Target Domain and Research Questions

Mathematics is a common thread across science, technology, engineering and mathematics (STEM) disciplines (Fitzallen, 2015). We examined word problems that include within-domain word problems in mathematics curriculum as well as between-domain word problems in STEM curriculum. For Studies 1 and 2, we focused on between-domain word problems in a chemistry context (Ngu et al., 2009; Ngu and Yeung, 2013). The target domain is the molarity chemistry word problems. For Studies 3, 4 and 5, we focused on within-domain word problems in mathematics context—the percentage change word problems (Ngu et al., 2014, 2016, 2018). While each study involved a comparison of different instructional approaches, we focused on the algebra approach that requires the integration of relevant information in an equation to solve a category of word problems that shares a similar problem structure. Of the five studies, four have diagrams to represent word problems. Our main aim was to examine the effect of the algebra approach for acquiring problem-solving expertise. Moreover, we included participants from either Asia (Studies 1, 2 and 4) or Australia (Study 3) or both Asia and Australia (Study 5). The purpose was to examine differential development of problem-solving expertise for word problems across different cultural settings. Specifically, we addressed two research questions:

1. *Are there a proportion of students who acquire expertise for solving word problems after studying worked examples?*
2. *Is there differential development of problem-solving expertise for word problems between Australian students and Malaysian students in regard to the equation approach (algebra approach)?*

### Study 1

**Figure 1:**
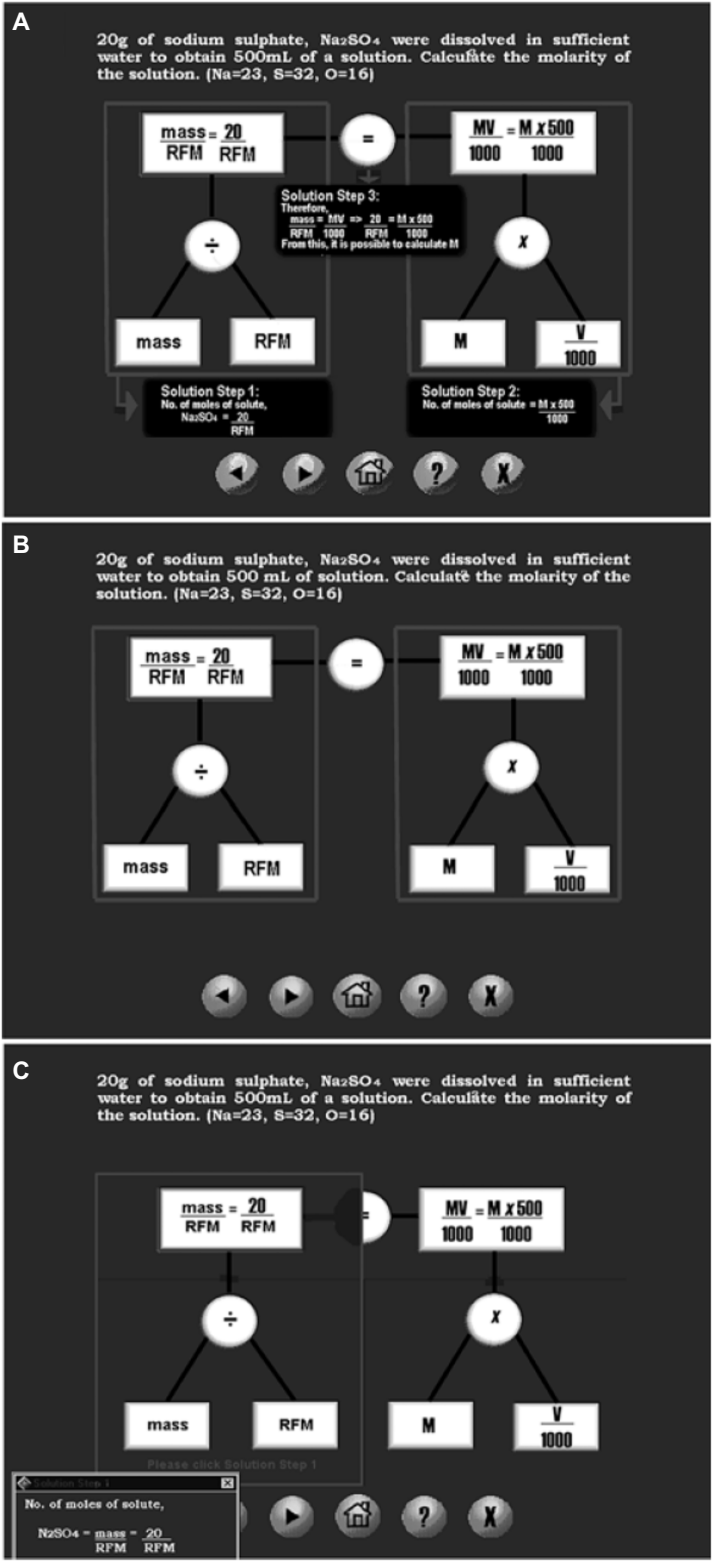
A worked example of a molarity problem: **(A)** static-solution format, **(B)** no-solution format, and **(C)** interactive-solution format. Source: Ngu et al. (2009).

The Study 1 aimed to facilitate students’ learning of molarity problems, which is a type of word problems in a chemistry context (Ngu et al., 2009). The Study 1 compared three computer-based formats for learning molarity problems: (a) static-solution format, (b) no-solution format, and (c) interactive-solution format (Figure 1) The design of the three computer-based format was based on the hierarchical network problem representation proposed by Nathan et al. (1992). The strength of the network problem representation depends on its ability to depict a hierarchical level of concepts (values, variable) and their relation without its irrelevant cover story. In essence, it allows the learner to visualize how the values, variable and their relation can be integrated in an equation, such as mass/RFM = MV/1000 for solution. It should be noted that the equation, such as mass/RFM = MV/1000, represents the problem structure of molarity problems. Thus, the conceptual knowledge of molarity problems was scaffolded by a hierarchical level of concepts (values, variable) and their relation, whereas its procedural knowledge was revealed in the solution steps.

The three computer-based formats differ in the design of the solution steps. For the static-solution format, all three solution steps are placed at relevant positions in the diagram, which eliminates a split-attention effect (Tarmizi and Sweller, 1988). For the no-solution format, no solution steps are provided. The cognitive load involved in deducing the solution steps would be low, given that the diagram explicitly displays the problem structure in which the solution steps are embedded. For the interactive-solution format, learners can learn from the diagram in three ways: (i) click relevant positions and have all three solution steps continuously—static-solution format, (ii) do not click the diagram—no-solution format, and (iii) click a relevant position to view one solution step at a time. The availability of different options to explore the diagram would expect to impose high cognitive load. Thus, it was hypothesized that the static-solution format would be better than the no-solution format, which in turn, would be better than the interactive-solution format.

We implemented a pre-test—intervention—post-test design. The pre-test shared identical content as the post-test and it provided a baseline score for students. The pre-test (or post-test) consisted of 5 similar problems and 4 transfer problems. The similar problems were isomorphic to the acquisition problems because both shared the same solution procedure (problem structure), whereas an adaptation of the solution procedure was required to solve transfer problems. The means for the pre-test ranged from 4 to 9% for the similar problems and 0 to 7% for the transfer problems. Forty-two Asian students aged about 15 years old from a secondary school participated in the study. A chemistry teacher introduced pre-requisite knowledge of molarity problems (e.g., molar mass and atomic mass) a week prior to the computer session. On the day of testing, all students completed a pre-test. Then, they were randomly assigned to the interactive-solution format (14 students), the static-solution format (14 students) and the no-solution format (14 students). Students across the three formats completed the computer session (25 min) in a laboratory where a computer was assigned to each student. First, they studied a brief instruction in regard to the use of the computer (e.g., use of icons and menu). Second, they studied an instruction sheet which provided the definition of molarity and two worked examples showing how to solve molarity problems. Third, students across the three formats studied (and not solved) eight molarity problems with the aid of a computer. According to Paas and Van Merriënboer (1994), studying worked examples only may potentially eliminate the negative effect caused by students attending to incorrect solutions generated, which could interfere with learning. Lastly, all students undertook a post-test. Students might learn from studying the instruction sheet; however, we expected the dominant learning to occur when they studied multiple worked examples with the aid of a computer.

One way ANOVA performed on similar problems revealed a significant difference between the three formats, *F*(2, 39) = 4.06, *p* = 0.03. A follow-up Tukey test indicated that the difference was between no-solution format (*M* = 0.36, SD = 0.41) and interactive-format (*M* = 0.06, SD = 0.12) where *p* = 0.025. Again, one way ANOVA performed on transfer problems showed a significant difference among the three formats, *F*(2, 39) = 5.48, *p* = 0.00. A follow-up Tukey test revealed that the difference was between static-solution format (*M* = 0.43, SD = 0.33) and interactive-solution format (*M* = 0.07, SD = 0.15) where *p* = 0.006. Overall, the results indicated that the no-solution format or static-solution format outperformed the interactive-solution format for molarity problems across the similar problems and transfer problems.

We computed the Relative condition efficiency, $E=\frac{P-M}{\sqrt{2}}$ where *E* = efficiency, *P* = performance and *M* = mental effort to examine the efficiency of the three instructional formats. The Relative condition efficiency attributes the performance outcomes to the cognitive load involved in processing instructional materials. One way ANOVA on *E* values was non-significant on similar problems, *F*(2, 39) = 2.08, *p* = 0.14, but it was significant on transfer problems at 10% level, *F*(2, 39) = 2.64, *p* = 0.08, indicating that the no-solution format was better than the static-solution format, which in turn was better than the interactive-solution format.

On examining students’ solution strategies, students in the interactive-solution format did not skip solution steps. However, six students (43%) from the static-solution format, and 8 students (57%) from the no-solution format skipped solution steps 1 and 2. They wrote mass/RFM = MV/1000 (first step), and substituted values and a variable (*M*) to solve the problem (second step; Table 1). The demonstration of a two-step strategy parallels prior studies of expertise development in problem-solving, whereby students could retrieve a single equation needed to solve molarity problems (e.g., Blessing and Ross, 1996). Presumably, the gaining of the conceptual knowledge had led to the execution of procedural knowledge efficiently, resulting in fewer solution steps.

**Table 1:** The Study, type of word problems, evidence of expertise, percentage of students demonstrated expertise development for similar problems.

Study	Word problems	Evidence of expertise	Instructional approaches		
Study 1 Ngu et al., 2009	Molarity chemistry problem: *20 g of sodium sulphate, Na_2_SO_4_ were dissolved in sufficient water to obtain 500 ml of a solution. Calculate the molarity of the solution. (Na = 23, S = 32, O = 16)*.	Step 1: mass/RFM = MV/1000 (where RFM is the relative formula mass). Step 2: substitute values and a variable and solve for M.	Interactive-solution format	Static-solution format	No-solution format
			–	43%	57%
Study 2 Ngu and Yeung, 2013	Same as Study 1.	Same as Study 1 except the use of “MM” instead of “RFM.” The “MM” means molar mass.	Text editing	Equation worked examples	–
			–	55%	
Study 3 Ngu et al., 2014	Percentage change problem: *If your father wants to increase your weekly allowance of $20 by 5%, what is your new allowance?*	Step 1: $20 + ($20 × 5%) Step 2: Calculate the new allowance.	Unitary approach	Pictorial approach	Equation approach
			–	–	82%
Study 4 Ngu et al., 2016	Same as Study 3.	(i) same as Study 3 (ii) wrote $20 × 5% and then added $20 to ($20 × 5%)	Unitary approach	Equation approach 48% for (i)	
			–	44% for (ii)	
Study 5 Ngu et al., 2018	Challenging percentage-change problem: *A shirt has been discounted 60% and now costs $80. What did it cost originally?*	Step 1: $80 = *x* – 60% *x* Step 2: solve the equation.	Unitary approach	Unitary–pictorial approach	Equation approach
			–	–	58%

*We only included data that are related to the use of the algebra approach for learning word problems*.

### Study 2

**Figure 2:**
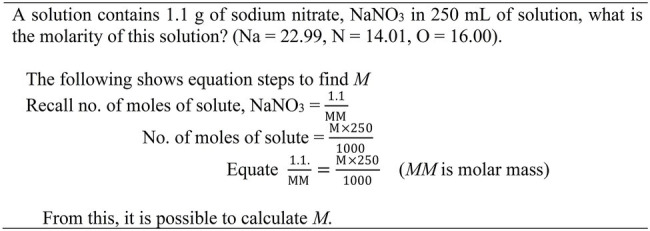
An equation worked example. Source: Ngu and Yeung (2013).

Once again, the objective of this Study 2 was to facilitate students’ learning of molarity chemistry problems (Ngu and Yeung, 2013). As indicated in Figure 2, the equation worked example consists of three equation steps. The emphasis is placed on the construction of an equation for solution (step 3). The design of the equation worked example does not represent the design of a typical worked example in which all solution steps to obtain a solution are provided. Instead, the equation worked example only portrays three key equation steps to solve molarity problems. It should be stressed that the three solution steps contain both conceptual knowledge and procedure knowledge for solving the molarity problems. The equation worked example was compared with the text editing condition. The text editing condition requires learners to scrutinize the problem text and indicate whether it contains missing, irrelevant or relevant information for solution. It places emphasis on identifying relevant information for solution; but, it falls short of addressing the procedural knowledge of solving the molarity problems.

The sample consisted of 22 Asian students aged about 17 years old from a secondary school. Students were randomly assigned to two groups: (i) text editing, and (ii) equation worked examples (Table 1). On Day 1, the chemistry teacher introduced pre-requisite knowledge (e.g., atomic mass and molar mass) pertaining to molarity problem. On Day 2, we implemented the experimental procedure which consisted of an acquisition phase and a test phase. We did not include a pre-test because all test materials and procedures had to align with the school curriculum. The post-test was similar to Study 1—it comprised 5 similar problems and 4 transfer problems. During the learning phase (20 min), students studied an instruction sheet and completed seven example–problem pairs. The instruction sheet provided the definition of molarity and a worked example illustrating how to solve a molarity problem. Again, the dominant learning would occur during which students completed multiple example-problem pairs rather than studying the instruction sheet. Each pair consisted of an equation worked example and a similar problem (Figure 2).

The equation worked examples group (*M* = 0.42, SD = 0.34) outperformed the text editing group (*M* = 0.16, SD = 0.25) on similar problems, *t*(20) = 2.00, *p* = 0.05. A significant difference between the equation worked example group (*M* = 0.45, SD = 0.31) and the text editing group (*M* = 0.14, SD = 0.26) was observed for transfer problems, *t*(20) = 2.60, *p* = 0.02. Thus, the results favored the equation worked examples group irrespective of similar problems or transfer problems.

Concerning the solution strategy, of those 11 students in the text editing group, only one demonstrated a two-step strategy (9%) typically shown by expert problem-solvers. However, six out of 11 students in the equation worked examples group demonstrated a two-step strategy (55%). Having acquired the schema for the molarity problem, students realized that they can skip intermediate steps 1 and 2. Hence, they retrieved the equation that integrated relevant information, mass/MM = MV/1000 (first step), and then substituted values and a variable to solve for *M* in step 3 (second step). Consistent with prior studies (Koedinger and Anderson, 1990; Blessing and Anderson, 1996), such step skipping performance to generate a two-step strategy indicates expertise development for molarity problems (Table 1). We attribute the development of expertise for molarity problems to the design of the equation worked examples that imposes low cognitive load. It is possible that the acquisition of conceptual knowledge of the underlying problem structure (mass/MM = MV/1000) facilitates the gaining of procedural knowledge, leading to the generation of reduced solution steps.

### Study 3

This Study 3 is related to learning how to solve percentage change problems, which is a type of word problems in everyday situations (Ngu et al., 2014). Sixty 8th grade Australian students (mean age = 14) were randomly assigned to: (i) unitary approach, (ii) pictorial approach, and (iii) equation approach (Table 1). Consider a percentage change problem used in the study, “*If your father wants to increase your weekly allowance of $20 by 5%, what is your new allowance?*.” Central to the unitary approach is the unit percentage concept. This unitary approach consists of three solution steps: (i) 100%+ 5% = 105% (increase by 5%), (ii) $20÷100 = $0.2 (calculate 1%), and (iii) $0.2 × 105 = $21 (calculate 105%). Each solution step cannot be understood independent of other solution steps. The interaction between elements within each solution step and across the three steps would constitute a high level of element interactivity and thus intrinsic cognitive load. Moreover, to calculate the sub-goal of the unit percentage (1%), the learner needs to integrate information from two separate sources [100% in (i) and $20 in (ii)], which will cause a split-attention effect (Lee and Kalyuga, 2011). Thus, the combined consequences of high level of element interactivity and extraneous cognitive load would render this approach ineffective.

The diagram of the pictorial approach aims at depicting the proportion concept—the alignment between quantity and percentage. A rectangular bar diagram is divided into 10 equal chunks and each chunk represents 10%. The alignment between quantity ($20) and percentage (100%) not only eliminates a split-attention effect (Lee and Kalyuga, 2011) but it also acts as a point of reference to calculate a sub-goal (quantity) that corresponds to 1, 5, 10%, etc. The learners need to learn the solution steps with reference to the diagram. Similar to the unitary approach, the learners need to process the interaction between multiple elements within each solution step as well as between the three solution steps. The germane cognitive load is increased to deduce the proportion of 10%:$2 and 5%:$1, leading to the calculation of new allowance, 105%:$21. Nonetheless, the pictorial approach may not be better than the unitary approach. The diagram would impose cognitive load when the learner needs to deduce a quantity that corresponds to % other than a multiple of 10% (e.g., 17%).

For the equation approach, similar to the schema-based instruction proposed by Jitendra et al. (2011), a diagram depicting a horizontal line is used to scaffold conceptual knowledge (problem structure) of percentage change problems. The germane cognitive load is increased to process the horizontal line, which aims at helping learners to translate the problem structure that consists of two components: (i) original allowance, and (ii) increased amount. In addition, the horizontal line also plays a crucial role in mapping the problem structure to an equation: New allowance = original allowance + increased amount. Within the topic of percentage problems, students would have learned percentage quantity prior to learning percentage change problems. Therefore, they were expected to process the increased amount, such as ($20 × 5%) as a single element. Overall, the processing of the equation approach entails the manipulation of two elements [$20 and ($20 × 5%)], which would impose low cognitive load. In other words, building on the prior knowledge of percentage quantity has lowered the intrinsic cognitive load of learning the percentage change problems. It was hypothesized that the equation approach would be better than the unitary and pictorial approach on learning how to solve percentage change problems.

**Figure 3:**
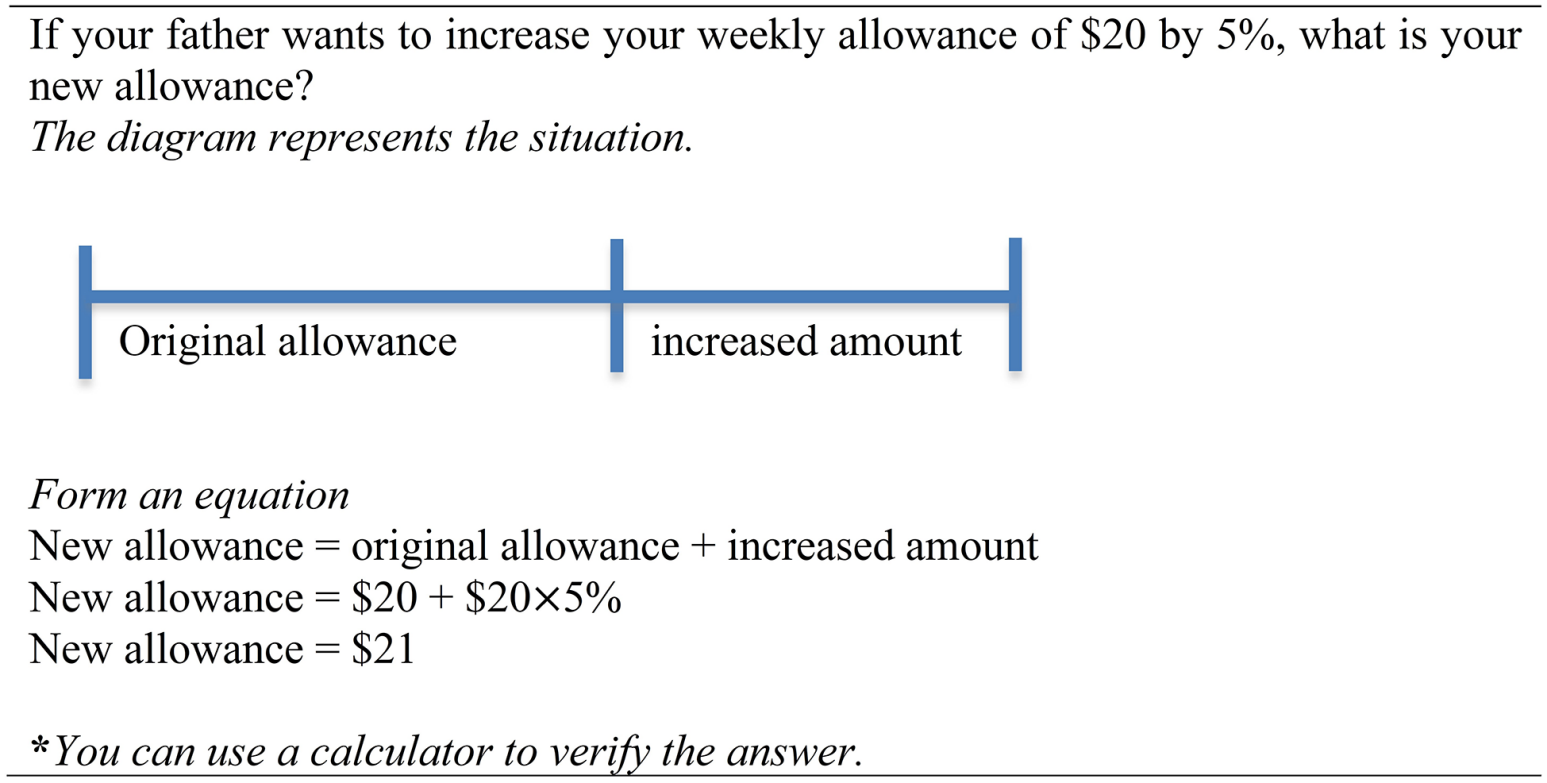
A worked example of the percentage change problem. Source: Ngu et al. (2014).

We conducted a pre-test—intervention—post-test design. Again, the pre-test which had similar content as the post-test served as a baseline to examine subsequent learning gain. The post-test consisted of 10 similar problems and 3 transfer problems. The means for the pre-test ranged from 7 to 17% for the similar problems and 0 to 2% for the transfer problems. The learning phase required students to study an instruction sheet and complete six example–problem pairs that took 20 min. The instruction sheet provided the definition of percentage, the review of the percentage quantity and a worked example showing how to solve a percentage increased problem. Each example–problem pair consisted of a worked example and an isomorphic problem (Figure 3). Once again, students might benefit from studying the instruction sheet, but learning was expected to occur predominately *via* the completion of multiple example–problem pairs.

Students who scored 80% or above in the pre-test and those who did not complete all test materials were excluded from the final data analysis. One-way ANOVA performed on similar problems showed significant difference between the three groups, *F*(2, 52) = 10.88, *p* = 0.01. *Post-hoc* Tukey test indicated significant differences between the unitary approach (*M* = 0.57, SD = 0.43) and pictorial approach, (*M* = 0.29, SD = 0.36) where *p* = 0.04; and also between the equation approach (*M* = 0.85, SD = 0.25) and pictorial approach, (*M* = 0.29, SD = 0.36) where *p* = 0.01; but non-significant difference between the unitary approach (*M* = 0.57, SD = 0.43) and equation approach (*M* = 0.85, SD = 0.25) was found.

Similarly, one-way ANOVA performed on transfer problems indicated significant difference between the three groups, *F*(2, 52) = 8.83, *p* = 0.01. Furthermore, *Post-hoc* Tukey test revealed significant differences between the equation approach (*M* = 0.58, SD = 0.43) and unitary approach (*M* = 0.19, SD = 0.36) where *p* = 0.01; and between the equation approach (*M* = 0.58, SD = 0.43) and pictorial approach (*M* = 0.11, SD = 0.27) where *p* = 0.01. Overall, the equation approach was better than the pictorial approach for the similar problems, and the other two approaches for the transfer problems.

**Figure 4:**
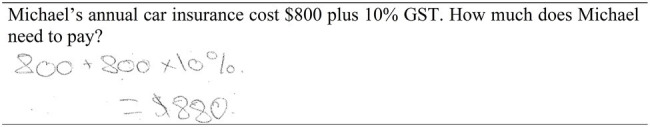
A solution of a percentage increased problem that shows step skipping performance.

In regard to the solution strategy, of those 17 students who received the equation approach, 14 could (82%) integrate relevant information in a single equation (first step) and calculate the answer (second step; Table 1). Once again, Study 3 shows the power of worked examples to assist students to develop expertise in which they not only identified the problem structure and used an equation to solve the problems, but also exhibited step skipping performance which is consistent with prior research (Koedinger and Anderson, 1990; Blessing and Anderson, 1996). We attribute the generation of a two-step strategy (Figure 4) as a result of the scaffold provided by the diagram that facilitates in-depth understanding of conceptual knowledge of the percentage change problems, and the solution steps that illustrate its procedure knowledge.

### Study 4

The target domain for Study 4 was the percentage change problems, which are similar to Study 3. Fifty-nine Asian students were randomly assigned to either the unitary approach or the equation approach. The design of the unitary approach and equation approach is the same as in Study 3. The research design involved a learning phase and a test phase. Again, we were unable to include a pre-test owing to the need to align the testing materials with the school curriculum. We focused on the equation approach (Table 1). The equation approach was similar to the equation approach in Study 3.

Data analysis was based on 57 students who completed all test materials. The equation group (*M* = 0.78, SD = 0.18) outperformed the unitary group (*M* = 0.67, SD = 0.22) on similar problems, *t*(55) = 2.01, *p* = 0.05. The equation group (*M* = 0.38, SD = 0.40) was marginally better than the unitary group (*M* = 0.22, SD = 0.29) for the transfer problems, *t*(55) = 1.74, *p* = 0.09. The unitary approach imposed significantly higher mental effort than the equation approach, *t*(55) = 2.76, *p* = 0.008, *r* = 0.35 (a medium effect). In addition, using the relative condition efficiency, $E=\frac{P-M}{\sqrt{2}}$ , the equation approach was significantly more efficient than the unitary approach, *t*(55) = 2.83, *p* = 0.006, *r* = 0.36 (a large effect).

On examining students’ solution strategies, 48% wrote a single equation, for example, 88 + (80 × 10%) (first step), and calculate the answer (second step). In addition, 44% wrote percentage quantity, for example, (80 × 10%; first step), and then added 80 to (80 × 10%) (second step). Once again, the success of the equation approach is clearly seen in students’ solution strategies. Hence, an in-depth understanding of the conceptual knowledge might contribute to the superior performance of procedural knowledge manifested in the reduced solution steps. Moreover, the robustness of the equation approach was confirmed in Asian context. It should be noted that 37% of Malaysian students in the unitary approach who did not have access to the equation approach used a modified version of the equation approach (two-step equation approach), such as (i) $250 × 12% = $30 (Step 1), and (ii) $250 + $30 = $280 (Step 2). Indirectly, this implies that Malaysian students may have stronger foundation than Australian students in Study 3 in regard to the use of algebra for solving word problems.

### Study 5

This Study 5 documented how to solve challenging percentage-change problems, which poses a challenge to students because the goal is to find the original quantity after a change of its original quantity (Parker and Leinhardt, 1995; Ngu et al., 2018). An example of a challenging percentage-change problem is shown in Figure 4: *The sale price of an item including a 10% Good and Service Tax (GST) is $264. Find the price of the item excluding GST*. The Australian sample (55 students, mean age = 16), and the Malaysian sample (75 students, mean age = 16) participated in the study. Students in each sample were randomly assigned to three groups (equation, unitary, unitary–equation approaches; Table 1).

**Figure 5:**
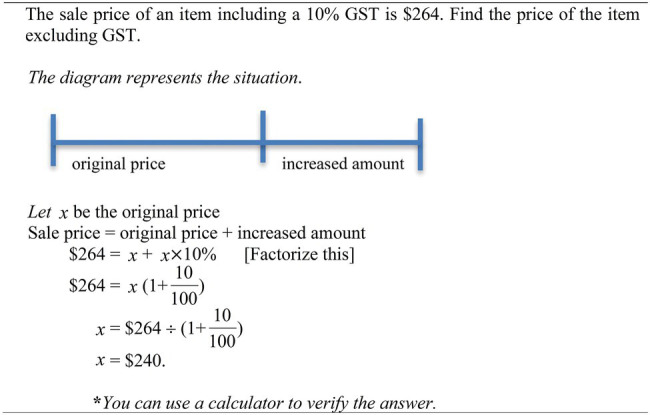
A worked example of a challenging percentage-change problem. Source: Ngu et al. (2018).

As shown in Figure 5 once again, the equation approach is accompanied by a horizontal line depicting two components: (i) original price, and (ii) increased amount. However, a variable, such as *x*, is used to denote the original price, and the increased amount of the original price (*x* × 10%). Again, the germane cognitive load is increased to process the problem structure portrayed in the horizontal line, resulting in the generation of an equation: Sale price = original price + increased amount. Similar to the Study 3 or Study 4, the diagram aimed to uncover conceptual knowledge of the challenging percentage-change problems. The subsequent solution steps involve the substitution of values ($264, 10%), a variable (*x*) to form an equation, such as $264 = x+x × 10%, and solve for *x*.

The unitary approach shares similar design features as the unitary approach in Study 3 or Study 4. The main concept of this unitary approach is the unit percentage. The learners are required to calculate 1%, and then a multiple of 1% to obtain the answer. Basically, it comprises three solution steps: (i) 100% + 12% = 112% (markup 12%), (ii) $34 ÷112 = $0.3035 (calculate 1%), and (iii) $0.3035 *×* 100 = $30.35 (calculate 100% which is the original price). Similar to the Study 3 or Study 4, the need to integrate information from the first solution step (112%) and the second solution step ($34) will cause a split-attention effect. Thus, the use of working memory to deal with the split-attention effect as well as the high element interactivity arises from the interaction between multiple elements within and across the solution steps would hinder effective learning.

The main difference between the unitary–pictorial approach and the unitary approach is that the former has a diagram. The germane cognitive load is increased to process the diagram which depicts the proportion concept, aligning the quantity to its corresponding percentage. In particular, the alignment between $34 and 112% not only eliminates a split-attention, but also facilitates the calculation of a subgoal (1%). Hence, the unitary–pictorial would impose lower cognitive load than the unitary approach.

We used a pre-test—intervention—port-test design. The pre-test had the same number and type of questions as the post-test—it provided the baseline score to examine the impact of different instructional approaches upon learning to solve challenging percentage-change problems. The means pre-test score for Australian students ranged from 0–3%, and Malaysian students was 0%.

Again, the learning phase was similar to Study 3. Students were given 20 min to study an instruction sheet and complete six example–problem pairs. The instruction sheet provided two worked examples, one of which was percentage increased problem and the other was percentage decreased problem. For each example–problem pair, they studied a worked example (Figure 5) and solve a similar problem. Once again, the main learning was expected to occur when students completed the example–problem pairs though they may also benefit from studying the instruction sheet.

Students who scored 80% or above in the pre-test were excluded from the final data analysis. In relation to the post-test, Malaysian students (*M* = 0.36, SD = 0.23) outperformed Austrian students (*M* = 0.16, SD = 0.27) for the for the equation approach in line with the hypothesis, *t*(37) = 2.52, *p* = 0.02. The Australian students and Malaysian students did not differ on the unitary approach, (*Ms* = 0.23 versus 0.30), *t*(36) = 0.97, *p* = 0.34, nor on the unitary–pictorial approach, (*Ms* = 0.47 versus 0.40), *t*(42) = 0.86, *p* = 0.40. In contrast to the hypothesis, Australian students did not outperform Malaysian students for the unitary–pictorial approach. Using pairwise comparisons, for Australian students, the unitary–pictorial group outperformed both the unitary group (*p* = 0.02), and the equation group (*p* = 0.00). In contrast, no differences were observed between the three groups for Malaysian students.

We analyzed the number of students who used respective solution strategies across the unitary approach, unitary–pictorial approach and equation approach for the post-test. A chi-square test indicated significant differences favoring Malaysian students for the equation approach, *χ*^2^(1, *N* = 39) = 27.57, *p* < 0.001, the unitary approach, *χ*^2^(1, *N* = 38) = 5.43, *p* = 0.02, and the unitary–pictorial approach, *χ*^2^(1, *N* = 44) = 6.12, *p* = 0.01. However, for the unitary–pictorial approach, Australian and Malaysian students demonstrated step skipping performance in that they generated two-step strategy. For example, consider a test item: *A shirt has been discounted 60% and now costs $80. What did it cost originally?* By discarding the first step “40% represents $80,” students wrote two steps: (i) 80 ÷ 40 = 2, and (ii) 2 × 100 = $200. A chi-square test indicated no difference between Australian students and Malaysian student on two-step strategy, *χ*^2^(1, *N* = 44) = 0.86, *p* = 0.36.

**Figure 6:**
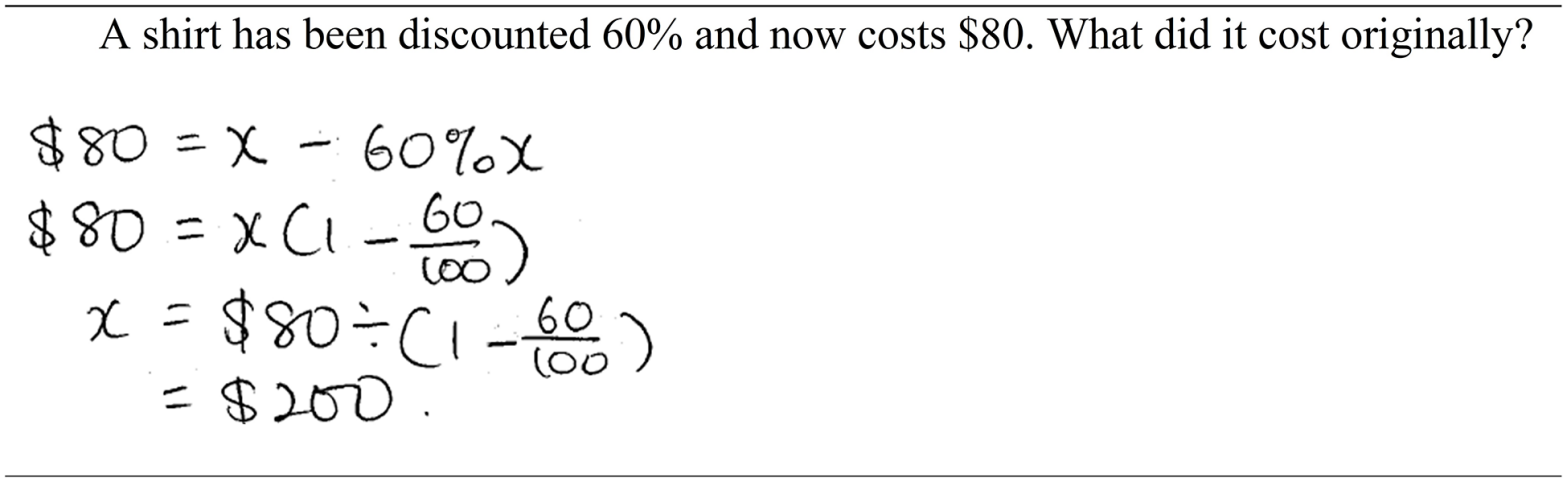
A solution of a challenging percentage decreased problem that shows step skipping performance.

Regarding the equation approach, importantly, 14 out of 24 (58%) Malaysian students in the post-test skipped some aspects of the solution procedure for the same test item above. They integrated relevant information and expressed it in an equation: $80 = *x* – 60%*x* (first step) and solve for *x* (second step; Figure 6). For Australian students, of those 5 students who provided accurate answers, none of them exhibited problem-solving expertise. In fact, many Australian students struggled with algebra (e.g., equation solving skills) and thus did not benefit from the equation approach. It is possible that Australian students could benefit from an alternative algebra approach that splits the solution procedure in two stages. In stage 1, the learner calculates % after a discount of 60%, which is 40%. In stage 2, the learner forms an equation, such as 40% *x =* $80, solve for *x*. This alternative approach may be easier because the variable (*x*) appears only once instead of twice ($80 = *x* – 60%*x*) in the equation (Koedinger et al., 2008).

Taken together, the results from Study 4 and Study 5 suggests that Malaysian students were more competent in using the algebra approach for solving word problems. The Australian students were not inferior to Malaysian students in regard to the use of non-algebra approach (unitary–pictorial approach) for solving word problems. Consistent with the results obtained in Study 3 and Study 4, once students had gained expertise, they were capable of using a single equation to represent the problem structure for subsequent generation of a solution. Once again, this illustrates the power of worked examples to facilitate the acquisition of domain-specific knowledge related to challenging percentage-change problems, leading to the generation of reduced solution steps.

## Discussion

This paper is primarily concerned with the review of five prior studies that documented the power of worked examples to help students in developing expertise for word problems across Mathematics and Science curriculum. The review has provided an answer to the research questions. Across five studies, approximately 50% of students who were exposed to the worked examples approach has acquired problem-solving expertise for word problems. The implementation of studying worked examples only (Study 1), and example–problem pairs (Studies 2, 3, 4, and 5) imposes relatively low cognitive load, thus facilitating the acquisition of problem-solving expertise. As summarized in Table 1, across five studies, students could generate an equation based on the information provided in the problem text (first step), and then solve the problem (second step). The demonstration of step skipping performance reflects the availability of a schema for a category of word problems, which encompasses conceptual knowledge and procedural knowledge. It appears that the presence of conceptual knowledge enhances the execution of procedural fluency in the form of fewer solution steps. In relation to cross-cultural comparison, Malaysian students outperformed Australian students for the equation approach (algebra approach) but not the unitary–pictorial approach (non-algebra approach). Presumably, such phenomenon is due to a stronger algebra foundation for Malaysian students.

### Theoretical Considerations

Research has indicated the benefit of providing of a diagram to enhance learning of word problems in the domain of probability (Schwonke et al., 2009). This is particularly the case when learners were informed that the function of a diagram aimed at bridging the relation between problem text and the equation. Thus, the provision of a diagram across Studies 1, 3, 4, and 5 that scaffolds the problem structure plays a critical role in developing problem-solving expertise. More specifically, in Study 1, studying the diagram that displayed a hierarchical order of the relation between individual structural elements had provided insights into the formation of an equation, leading to the generation of a two-step strategy. In Studies 3, 4 and 5, a horizontal line was divided into two different lengths that corresponded to two different quantities (e.g., an increased amount as a fraction of the whole amount), thus scaffolding the conceptual knowledge of percentage change problems. Such a display of visual information assisted students to formulate an equation to solve percentage problems efficiently. While Study 2 did not have a diagram, an emphasis of having three key solution steps that encompass both conceptual knowledge and procedural knowledge allowed students to infer that the third equation step was a critical step that contained the problem structure expressed in an equation for solution. On the other hand, without the aid of a diagram to scaffold the problem structure (such as the proportion concept in the unitary approach) across Study 3–5, learners struggled to acquire skills for solving word problems.

Previous research has focused on analogical learning to facilitate the acquisition of schema for word problems (Reed, 1989). It highlights the mapping of concepts between two problems that share a similar problem structure. However, it falls short of providing instructional support to integrate relevant information and express this in an equation. In contrast, a worked example accompanied by a diagram is effective, because the diagram provides clues to organize problem structure from the problem text and express this in an equation. In addition, completing multiple example–problem pairs enables students to gain familiarity with a category of word problems that shares a similar problem structure and thus a similar solution procedure. Accordingly, students developed problem–solving expertise for word problems as a result of exposing to worked examples.

### Practical Implication for Mathematics Education

The Australian educators are encouraged to consider the merit of cognitive load theory in designing instructions for effective learning (NSW Education: Centre for Education Statistics and Evaluation, 2017). The current popular mathematics textbooks (e.g., Vincent et al., 2012) do not include the use of worked examples especially the implementation of example–problem pairs to enhance mathematics learning. Hence, it is timely to promote greater use of worked examples to develop problem-solving expertise especially for word problems that presents a challenge to students.

The use of worked examples to facilitate expertise development also depends on students’ prior knowledge (Kalyuga et al., 2003). For example, students who are weak in basic algebra concepts, such as the meaning of variable, factorization, and equation solving skills, may not benefit from the equation approach for learning challenging percentage problems, let alone develop expertise for this type of problems. Thus, it is important to strengthen students’ prior knowledge of algebra before exposing them to the use of the algebra approach for learning word problems. Furthermore, having prior knowledge of equivalent fractions (e.g., 1/10 = 10/100) would have facilitated the processing of the proportion concept in a diagram (e.g., unitary–pictorial approach) with fewer elements (Carlson et al., 2003).

### Limitations and Future Directions

This review highlights the importance of using the algebra approach (i.e., the equation approach) to facilitate the acquisition of problem-solving expertise for word problems. However, there are other instructional approaches which could have achieved the same purpose. For example, in Study 5, the unitary–pictorial approach had also shown to be effective for challenging percentage-change problems. The diagram in the unitary–pictorial approach scaffolded the relation between quantity and percentage based on proportional reasoning. Consequently, students demonstrated step skipping performance, which was reflected in their solution strategies. Thus, future review should explore the design of different worked example formats that has the potential to facilitate the development of problem-solving expertise.

Early work by Cooper and Sweller (1987) indicates the importance of an extended practice time to enable students to automate the schema to solve not only similar problems but also transfer problems that require the adaptation of the solution procedure. Students across the five studies demonstrated step skipping performance despite a relatively short learning phase of about 25 min. Nonetheless, future research should provide a longer learning phase to enable students to develop not only expertise in solving similar problems, but also skills to solve transfer problems. In addition, a longer learning phase would also allow more students (e.g., more than 50%) to develop expertise for solving word problems.

Across the five case studies, the use of worked examples has facilitated the development of problem-solving expertise for a category of word problems sharing the same schema. Mayer (1982) distinguished standard problems (similar problems) and non-standard problems (transfer problems). Mayer regarded standard problems as a category of word problems that share the same schema. Unlike standard problems, non-standard problems do not share the same schema. In fact, an adaptation of the solution procedure for solving standard problems is required to solve non-standard problems. Overall, the strength of worked examples lie in its ability to assist learners to develop expertise for solving similar problems (standard problems), and to a lesser extent, transfer problems (non-standard problems).

Prior studies have uncovered the challenge for problem-solvers to interpret differences in syntax (word order) within specific type of word problems. Clement (1982) found that students tended to interpret the syntax of word problems in a static rather than relational manner. They tended to produce 6S = P for a statement “There are six times as many students as professors at this university.” In a related study, successful problem-solvers were capable of using relational key words (e.g., less) to form an equation accurately, whereas unsuccessful problem-solvers interpreted the syntax literally, leading to an incorrect equation (Hegarty et al., 1995). In fact, the main issue here is failure for problem-solvers to interpret relational variables which stand for variables rather than objects. Strengthening learners’ prior knowledge of the concept of variable would alleviate learners’ working memory capacities for processing numerical and translation dimensions of word problems. Additional research is needed to verify this proposition.

## Conclusion

Drawing on the review of the five studies, the main ideas and interpretations presented pointing to the efficacy of studying worked examples alone or completing multiple example–problem pairs to develop problem-solving expertise for word problems. A typical worked example provides a diagram that scaffolds conceptual knowledge (problem structure) and procedural knowledge (solution steps). There is evidence that an in-depth understanding of conceptual knowledge (i.e., problem structure) might contribute to superior procedural fluency manifested in the reduced solution steps. Thus, we urge mathematics educators to consider the incorporation of worked examples in mathematics classroom to assist students in gaining problem-solving expertise for word problems.

## Author Contributions

BN and HP contributed equally to the articulation, conceptualization, and write-up of this manuscript. All authors contributed to the article and approved the submitted version.

## Conflict of Interest

The authors declare that the research was conducted in the absence of any commercial or financial relationships that could be construed as a potential conflict of interest.

## Publisher’s Note

All claims expressed in this article are solely those of the authors and do not necessarily represent those of their affiliated organizations, or those of the publisher, the editors and the reviewers. Any product that may be evaluated in this article, or claim that may be made by its manufacturer, is not guaranteed or endorsed by the publisher.
